# *Grindelia squarrosa* Extract and Grindelic Acid Modulate Pro-inflammatory Functions of Respiratory Epithelium and Human Macrophages

**DOI:** 10.3389/fphar.2020.534111

**Published:** 2021-01-18

**Authors:** Barbara Gierlikowska, Agnieszka Filipek, Wojciech Gierlikowski, Dominika Kania, Joanna Stefańska, Urszula Demkow, Anna K. Kiss

**Affiliations:** ^1^ Department of Laboratory Diagnostics and Clinical Immunology of Developmental Age, Medical University of Warsaw, Warsaw, Poland; ^2^ Department of Pharmacognosy and Molecular Basis of Phytotherapy, Medical University of Warsaw, Warsaw, Poland; ^3^ Department of Internal Medicine and Endocrinology, Medical University of Warsaw, Warsaw, Poland; ^4^ Department of Pharmaceutical Microbiology, Centre for Preclinical Research and Technology (CePT), Medical University of Warsaw, Warsaw, Poland

**Keywords:** Grindelia squarrosa, grindelic acid, inflammation, cold syndrome, respiratory epithelium, macrophages

## Abstract

**Aim of the study:** Both nasal and bronchial epithelial cells have evolved sophisticated mechanisms involved in cellular response to bacterial infection. Recognition of pathogens by TLR receptors activate the NF-κB transcription factor, and lead to production of wide spectrum of cytokines (TNF-α, IL-1β, IL-6 and IL-8). Released by epithelium proinflammatory cytokines intensify migration of macrophages to damaged tissues and modulate their pro-inflammatory functions. Based on traditional use of *G. squarrosa* aerial parts we hypothesized that successful treatment of cold-related diseases may arise from modulation of the pro-inflammatory functions of respiratory epithelium and human monocytes/macrophages. The biological activity of *G. squarrosa* extract and grindelic acid were compared with clarithromycin and budesonide used as positive controls.

**Methods:** The expression of surface receptors (TLR-4, IL-10) and expression of adhesive molecules (ICAM-1, VCAM-1, E-selectin) was analyzed with flow cytometry. The macrophage attachment to the epithelial cells was assessed fluorimetrically. The p65 NF-κB concentration and cytokine production was measured spectrophotometrically using enzyme-linked immunosorbent assay. Antibacterial activity was examined by the standard disc-diffusion method and serial dilution method according to CLSI guidelines.

**Results:** *G. squarrosa* extract and grindelic acid had no antimicrobial effect. However, we noticed significant modulation of pro-inflammatory functions of LPS-stimulated nasal and bronchial epithelium. *G. squarrosa* extract treatment resulted in decrease of TLR-4 expression and p65 NF-κB concentration and inhibition of cytokines synthesis (IL-8, TNF-α, IL-1β and IL-6) in both cellular models. Additionally, *G. squarrosa* extract slightly modulated ICAM-1 expression affecting on attachment of macrophages to epithelium. Only *G. squarrosa* extract was able to stimulate the anti-inflammatory functions of macrophages by inducing TGF-β release and IL-10 receptor surface expression. Grindelic acid, identified as a dominant compound in the plant extract, modulated pro-inflammatory functions of epithelium and macrophages slightly.

**Conclusion:** The obtained results support traditional use of *Grindelia squarrosa* preparations for a treatment cold-associated diseases symptoms. In our opinion, the observed biological effect of extract may be a consequence of synergistic effect of all compounds present in the extract.

## Introduction

Common cold is one of the most common occurring acute illnesses in humans. In a large-scale survey, 25% of all those questioned in the United Kingdom had suffered from 3 to 6 colds during the previous year and 73% reported having a common cold at least once during this period. The similar tendency is observed in Germany and United States. The acute episode of a cold is starting with viral infection and may be accompanied by bacterial superinfection. The most common bacterial isolates from people with cold syndromes include Gram-positive strains, such as *Streptococcus pneumoniae* responsible for pharyngitis, bronchitis or pneumonia (Klugman and Feldman, 2001); *Staphylococcus aureus* responsible for pharyngitis and epiglottitis, *Hemophilus influenzae*, which occupy a similar microenvironment within the nasopharynx as *Streptococcus pneumoniae* (Erwin and Smith, 2007)*,* and other opportunistic organisms as *Escherichia coli* (Mizgerd, 2008).

Common cold treatment is mostly directed towards relief of symptoms. The infection results in development of full-symptomatic inflammation and overproduction of cytokines. Currently accepted cold treatment strategies focus on suppression of inflammation by anti-inflammatory drugs. Treatment may be enhanced by inclusion of antibiotic therapy. However, using antibiotics bring benefits only for the treatment of secondary bacterial infections. The valuable approach in the cold treatment may be strengthen immune system in order to prevent the uncontrolled progression of inflammation, but also protection of the respiratory epithelium against excessive damage.

Because airway epithelial cells are a border line between external and internal environment, they are particularly vulnerable to damage. Epithelial cells express a wide spectrum of receptors, such as Toll-like receptors. TLRs identify pathogen-associated molecular patterns (PAMPs), and initiate the appropriate cellular response. Among known TLRs, TLR-4 is the main LPS receptor (Zusso et al., 2019). Binding the LPS results in the activation of downstream mediators, including the transcription factor nuclear factor NF-κB, which stimulates the production of pro-inflammatory cytokines (IL-1β, TNF-α and IL-6) (Spiegler et al., 2020). Released cytokines intensify migration of macrophages to the site of infection. The released cytokines modulate adhesion molecules expression (ICAM-1, VCAM-1 and E-selectin) on the respiratory epithelium, thus mediate direct emigration of macrophages across the endothelial and epithelial barriers that separate the bloodstream from the pulmonary air spaces (Martin et al., 1992; Martin, 2000; Puneet et al., 2005). The infiltrated macrophages are activated and released wide spectrum of inflammatory mediators: reactive oxygen species (ROS), cytokines (TNF-α, IL-6, TGF-β), and modulate expression of membrane receptors such as IL-10 (Michalak et al., 2018).

According to World Health Organization (WHO) guidelines, over 90% of the population of developed and developing countries, uses the wealth of phytomedicine. *Grindelia squarrosa* well-known in South America, Asia and Europe is still sold as complex preparations (liquid extracts, syrups) for treating cough and bronchitis (Strzelecka, 2000). According to European Medicines Agency (EMA) there are various types of preparations: tincture or herbal tea (Table 1). The toxicity of this plant to humans is negligible, what intensified the traditional use of the herb (Goetz, 2005).

**TABLE 1:** The forms of *G. squarrosa* extract administration.

Form	Administration	Part	References
Infusion	3 g in 150 ml of boiling water. Daily dose: up to 3 times daily	Aerial parts, flowers, leaves	Kalktenbach and Schimmer (1991), Ferreres et al. (2014)
Tincture	0.5–1 ml 3 times daily. Daily dose: 1.5–3 ml	Aerial parts, flowers	Kalktenbach and Schimmer (1991)
Liquid extract	0.6–1.2 ml 3 times daily. Daily dose: 1.8–3.6 ml	Aerial parts, flowers, leaves	Kalktenbach and Schimmer (1991), McFarlane (2017)

The *Grindelia* is valuable source of biologically active compounds (Capasso et al., 2003). The aerial parts of *G. squarrosa* are a rich source of diterpenes. It contains grindelic, 6-oxygrindelic, 18-hydroxy-6-oxygrindelic, 7-alpha-oxodihydrogrindelic and 8-alpha-oxodihydrogrindelic acids. As dominant diterpene was identified grindelic acid, which may consist of 20–60% of grindelia resin and up to 6% of dry plant material (Johnson, 1887; Efferth and Koch, 2011; Nowak and Rychlińska, 2012; Veres et al., 2014). The plant also contains flavonoids, phenolic acids, an essential oils, polyacetylenes and saponins (Veres et al., 2014).

Although *G. squarrosa* extracts are present at pharmaceutical market, there is still lack of studies fully explaining their anti-microbial and anti-inflammatory properties. In present study, we have evaluated influence of *Grindelia squarrosa* extract on pro-inflammatory functions of LPS-stimulated nasal and bronchial epithelial cells and macrophages. We highlight new anti-inflammatory properties of the extract, and explain how *G. squarrsa* extract modulates the epithelium response to LPS.

## Materials and Methods

### Chemicals

Normal Human Bronchial Epithelial Cells (NHBE) and Bronchial Epithelial Cell Growth Medium were obtained from Lonza (Switzerland). Primary Human Nasal Epithelial Cells (HNEpC) and Airway Epithelial Cell Growth Medium were obtained from Promocell (Germany). Grindelic acid (purity ≥95% (LC/MS-ELSD)), clarithromycin (purity ≥95% (HPLC)), budesonide (purity ≥98% (HPLC)), LPS (from *Escherichia coli* 0,111:B4), Ficoll Hypaque gradient, GM-CSF, HEPES buffer, l-glutamine, RPMI 1640 medium, Fetal bovine serum (FBS), Cell Dissociation Solution (non-enzymatic), Accutase™, propidium iodide and Calcein-AM were purchased from Sigma-Aldrich Chemie GmbH (Steinheim, Germany). TLR-4-(PE)-conjugate (cat no. HTA125) was obtained from eBioscience. Human Quantikine ELISA Kits were purchased from R&D System (Minneapolis, USA). Anti-Human CD54-(-APC) (cat no. 559771), Anti-Human CD106-(-PE) (cat no. 555647), Anti-Human CD62E-(-PE) (cat no. 551145) were purchased from BD Pharmingen.

### Plant Material and Phytochemical Analysis

The plant material *Grindelia squarrosa* (Pursh) Dunal (http://www.theplantlist.org/tpl1.1/record/gcc-87994) was collected in July 2018 in Lublin Medicinal Plant Garden, Department of Pharmacognosy with Medicinal Plant Unit, Medical University of Lublin (51°15′22″N, 22°33′51″E) were the voucher specimen is deposited (27a/13).

The air-dried plant material (5 g) was extracted with 60% ethanol (v/v, 1:10) in a water bath (70 °C) for 1 h three times. Then, combined extracts were filtered, the ethanol was evaporated, and the water residue was lyophilized. The phytochemical characterization was performed using UHPLC-DAD-ESI-MS^n^ analysis (UHPLC-3000 RS system, Dionex, Germany) with DAD detection and an AmaZon SL ion trap mass spectrometer with an ESI interface (Bruker Daltonik GmbH, Bremen, Germany). Separation was performed on a Zorbax SB-C_18_ column (150 × 2.1 mm, 1.9 μm) (Agilent, Santa Clara, CA, United States). The mobile phase consisted of 0.1% HCOOH in water (A) and 0.1% HCOOH in MeCN (B) using the following gradient: 0–60 min 5–100% B. The LC eluate was introduced into the ESI interface and compounds were analyzed in negative ion mode (for phenolic acids and flavonoids) and with positive ion mode (for diterpenes) with the following settings: nebulizer pressure of 40 psi; drying gas flow rate of 9 L/min; nitrogen gas temperature of 300 °C; and a capillary voltage of 4.5 kV. The mass scan ranged from 100 to 2,200 m/z. UV spectra were recorded in the range of 190–350 nm. The characterization of substances in the extract was performed by comparing the retention times and spectra (UV, MS, MS^n^) with published data.

Isolation of grindelic acid (GA). The plant material (50 g) was extracted three times with 60% ethanol (1:20) at 70 °C for 2 h each time. The ethanol from combined extracts was evaporated under reduced pressure, and the aqueous residue was then extracted four times with ethyl acetate (150 ml). The ethyl acetate residue (5.2 g) was subjected to Sephadex LH-20 column chromatography (80 × 2.5 cm) and eluted 70% MeOH to obtain four main fractions of 0.236 g (F1), 0.805 g (F2), 1.062 g (F3) and 0.480 g (F4), respectively. Fraction F4, containing GA, based on TLC analysis with comparison with standard, was separated on a Silica gel column (55 × 2.5 cm; 100–200 μm) with CHCL_3_-MeOH gradient (100:0 → 90:10) in seven steps. Ten fractions of 25 ml each were pooled into six main fractions (F4_1-F4_6) based on their TLC profiles. From fraction F4_1 (0.22 g) GA (152 mg), was isolated using Silica gel column (55 × 2.5 cm; 0.63 μm) and CHCL_3_ as eluent. The structure of GA was confirmed by their ^1^H and ^13^C NMR spectra, which were compared with reference data (Reta et al., 2013).

### Preparation of Solutions of Extract and Compounds for Bioassays

For all experiments *G. squarrosa* extract and isolated grindelic acid were used as described above. Extract was dissolved in DMSO (10 mg/ml stock solution) and tested at a concentration range 25–100 µL. Grindelic acid was dissolved in DMSO (10 mM stock solution) and tested at a concentration range of 10–50 µM. The final concentration of DMSO was adjusted to 0.1% (v/v) in culture media.

### Antimicrobial Properties of *G. squarrosa* Extract and Grindelic Acid

Both Gram-positive (*Streptococcus pneumoniae* ATCC 49619 and *Staphylococcus aureus* ATCC 25923 ATCC 29213) and Gram-negative (*Haemophilus influenzae* ATCC 49247) bacteria strains were tested in this study.

Sensitivity of selected bacteria strains was examined by the standard disc-diffusion method according to CLSI (previously NCCLS) guidelines (Watts, 2008). The results (diameter of the growth inhibition zone) were read after 18 h of incubation at 35 °C. Minimal Inhibitory Concentrations (MIC) were evaluated by the two fold serial microdilution method (in 96-well microtiter plates) using Mueller-Hinton Broth medium (Beckton Dickinson) according to CLSI guidelines (Bandi and Kompella, 2001; Watts, 2008).

### Primary Human Nasal Epithelial Cells (HNEpC) Culture and Stimulation

HNEpC cells (passage fourth to sixth), were seeded on 12-well plates, 1.5 × 10^5^ cells per well in Airway Epithelial Cell Growth Medium supplemented with 6% Fetal Bovine Serum. The confluent cells were treated with extracts/compounds and then used for experiments.

### Normal Human Bronchial Epithelial (NHBE) Culture and Stimulation

NHBE cells (passage fourth to sixth), were seeded on 12-well plates, 1 × 10^5^ cells per well in Bronchial Epithelial Cell Growth Medium. The confluent cells were treated with extracts/compounds and then used for experiments.

### Isolation of Human Monocytes and Stimulation

Monocytes were isolated immediately after collection using a Ficoll Hypaque gradient (Dudek et al., 2017). The mononuclear cell band was removed by aspiration, and cells were suspended in RPMI 1640 medium with 2 mM l-glutamine, 10 mM HEPES, antibiotics and autologous serum (5%). To promote the adherence of monocytes/macrophages, the peripheral blood mononuclear cell suspension was placed in 12-well tissue culture plates (2 × 10^6^ per well) and incubated for 2 h at 37 °C under humidified 5% CO_2_ air. After the incubation, nonadherent cells were removed, and adherent cells were cultured with RPMI 1640 medium supplemented with heat-inactivated fetal bovine serum (FBS, 10%) and granulocyte macrophage-colony stimulating factor (GM-CSF, 10 ng/ml) for 7 days to induce differentiation to macrophages. The medium and autologous serum were replaced every 2 days.

### Determination of Cytotoxicity by PI-Staining

Cytotoxicity of the tested extract and compounds was determined by PI-staining. Cells were treated with the compounds for 24 h, centrifuged (1,500 RPM; 5 min; 4 °C) and re-suspended in 300 µL of PBS. 5 µL of PI (50 μg/ml) solution was added to the cell suspensions and incubated 10 min in the dark. Cytotoxic effects of the extract and compounds were measured by BD FACS LSR Fortessa flow cytometer (BD Biosciences, San Jose, CA, United States) by recording 10,000 events per sample and analyzed with FlowJo V10 software. Cells that displayed high permeability to PI were expressed as percentage of PI(+) cells.

### Expression of TLR-4 Receptor on Nasal and Bronchial Epithelium

Influence of the tested extract and compounds on the TLR-4 receptor expression was determined by flow cytometry measurement. After 1 h extract- or compounds pretreatment cells were stimulated with LPS for 15 min, and centrifuged (1500 RPM; 10 min; 4 °C). After removing supernatants, cells were re-suspended in 300 μL PBS and labelled with monoclonal antibodies against TLR-4 (eBioscience, United States). The TLR-4 expression was measured by BD FACS LSR Fortessa flow cytometer (BD Biosciences, San Jose, CA, United States) by recording 10,000 cells per sample and analyzed with FlowJo V10 software.

### TNF-α, IL-1β, IL-6 and IL-8 Production

Epithelial cells were pretreated with the *G. squarrosa* extract (at concentration range 25–100 μg/ml) or grindelic acid (at 10–50 μM) for 1 h at 37 °C, and then they were stimulated with LPS (1 μg/ml) for 24 h. Afterwards, cells were centrifuged (1500 RPM; 10 min; 4 °C) and collected supernatants. The total amount of released cytokines was measured by enzyme-linked immunosorbent assay (ELISA) following the manufacturer’s instructions (BD Biosciences, United States). The results were expressed as percentages of the released agents from samples compared to the LPS-stimulated control.

### p65 NF-κB Concentration in Nasal and Bronchial Epithelium

Epithelial cells were pretreated with the *G. squarrosa* extract (at concentration range 25–100 μg/ml) or grindelic acid (at 10–50 μM) for 40 min at 37 °C, and then they were stimulated with LPS (1 μg/ml) for 20 min. The protein isolation was provided as we describe previously (Gierlikowska et al., 2020b). Cells were washed with ice-cold PBS, suspended in hypotonic buffer (5 mM NaF, 0.1 mM EDTA, 20 mM HEPES, 10 µM Na_2_MoO_4_), and then centrifuged. The nuclear pellets were lysed with freshly-prepared buffer supplemented with protease inhibitor cocktail and left on ice for 30 min. Pellets were centrifuged for 15 min 2.500 RPM and kept at −80 °C for further analysis.

The protein concentration was measured with Bradford-based method following manufacturer’s protocol. 10 µg of nuclear fraction was transferred on 96-well plate provided by manufacturer. Then, primary antibody (1:1,000 dilution) was added on plate for 1 h, then washed 3 times and incubated with HRP-conjugated secondary antibody (1:1,000 dilution) for additional 1 h. After incubation, plate was washed 3 times and reaction was stopped by adding developing solution and stop solution, respectively. The concentration of NF-κB p65 was measured spectrophotometrically at 450 nm.

### Expression of ICAM, VCAM and E-Selectin on Respiratory Surface

Cells were pre-incubated with extract or compounds for 1 h and then stimulated with LPS (1,000 ng/ml, for 24 h). Cell monolayers were washed with PBS and suspended in 200 μL of accutase. After 10 min incubation at 37 °C, the cells were harvested, centrifuged (1500 RPM; 10 min; 4 °C) and suspended in 300 µL of PBS. Cells were labelled with 5 μL of anti-human CD54 (ICAM-1), anti-human CD106 (VCAM-1) or anti-human CD62E (E-selectin).

The expression of adhesive molecules was analyzed by BD FACS LSR Fortessa flow cytometer (BD Biosciences, San Jose, CA, United States) by recording 10,000 cells per sample and analyzed with FlowJo V10 software. The effect on the surface expression of adhesion molecules was evaluated based on a software-generated marker histogram M1 for LPS stimulated cells.

### The Attachment of Macrophages to Epithelial Cells

Epithelial cells were pretreated with extract or compounds for 1 h and then stimulated with LPS (1,000 ng/ml, for 24 h). Cell monolayers were washed, suspended in 200 μL of RPMI 1640 medium and added 100 μL of 1 mM calcein-AM labelled macrophages (1×10^6^) as described previously (Michalak et al., 2018). Before calcein labelling, macrophages were stimulated with LPS (100 ng/ml) for an 30 min, and then incubated with epithelial cells for additional 30 min. The cell monolayers with attached macrophages were lysed with 0.1% Triton X-100, and fluorescence was measured in a microplate reader at 485 nm excitation and 520 nm emission.

### Expression of IL-10 Receptor on the Macrophage Surface

Macrophages were stimulated with LPS (100 ng/ml for 1 h) and treated with the extract or compounds for 24 h. Subsequently, cells were centrifuged (13,000 RPM, 4 °C, 1 min), suspended in 300 μL of PBS and labelled with antibodies against IL-10. The IL-10 expression was measured by BD FACS LSR flow cytometer (BD Biosciences, San Jose, CA, United States) by recording 10,000 cells per sample and analyzed with CellQuest Pro software. The results were expressed as percentages of cells expressing IL-10 receptor in comparison to control cells stimulated by LPS.

### TGF-β, IL-6 and TNF-α Production by Macrophages

Macrophages were cultured in 12-well plates in the presence of LPS (100 ng/ml, 1 h) and then treated with tested extracts/compounds for 24 h at 37 °C with 5% CO_2_. After 24 h, cells were harvested and centrifuged (2,000 RPM; 10 min; 4 °C). The amounts of released cytokines were measured by ELISA following the manufacturer’s instructions (BD Biosciences, United States). The effect on cytokine production was calculated by comparing the percentages of the released agent to the control cells, which were stimulated but were not exposed to the test compounds.

### Statistical Analysis

The results were expressed as the mean ± SEM from three independent experiments assayed in triplicates. All analyses were performed using Statistica 13.1 software. GraphPad Prism (version 5.01) was used to plot data. The statistical significance of the differences between means was established by ANOVA with Dunnett’s *post hoc* test. *p* values below 0.05 were considered statistically significant.

## Results

### *G. squarrosa* Extract Phytochemical Characterization and Grindelic Acid Isolation

**FIGURE 1:**
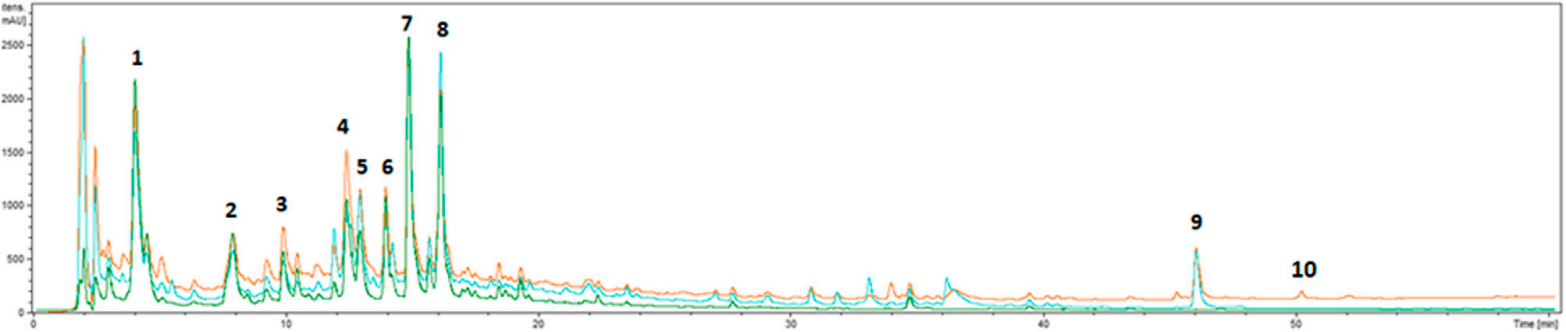
UHPLC-DAD chromatograms of extract from *Grindelia squarrosa* at 220 nm (orange line), 254 nm (blue line) and 325 nm (green line).

The phytochemical analysis of *G. squarrosa* extract was performed using a UHPLC-DAD-ESI-MS^n^ method. We confirmed the presence of polyphenolic compounds which are characteristic for Astearaceae family such as caffeoyl quinic acid and dicaffeoyl quinic acids, as well as luteolin and apigenin derivatives (Figure 1; Table 2). As for *Grindelia* genus the presence of diterpenes is distinctive, we were focused on this group of compounds. However, as grindelic acid do not possess chromophore groups, its detection based on UHPLC-DAD-ESI-MS^n^ was less sensitive. In order to confirm the presence of grindelic acid, we isolated this compound from the extract using ethyl acetate partition and column chromatography (Supplementary Figure S1). We confirmed the purity and structure of isolated GA using NMR spectroscopy (Supplementary Figure S2).

**TABLE 2:** Retention time, UV and MS data of the compounds present in extract from *Grindelia squarrosa*.

	Compound	Retention time [min]	UV [nm]	[M-H]^−^m/z	Fragment ion(s)	References
1	Caffeoyl quinic acid	4.0	325	353	191	Ferreres et al. (2014)
2	Feruloyl quinic acid	7.9	325	367	193	Ferreres et al. (2014)
3	Luteolin hexurosyl-pentoside	9.8	330	593	461, 417, 285	Ferreres et al. (2014)
4	Luteolin hexuronide	12.5	334	461	461, 285	Ferreres et al. (2014)
5	Apigenin hexurosyl-pentoside	12.9	334	577	445, 269	Ferreres et al. (2014)
6	Dicaffeoyl quinic acid (I)	14.0	325	515	353, 191, 179, 173	Ferreres et al. (2014)
7	Dicaffeoyl quinic acid (II)	14.9	325	515	353, 191, 179	Ferreres et al. (2014)
8	Dicaffeoyl quinic acid (III)	16.1	325	515	353, 191, 179, 173, 135	Ferreres et al. (2014)
9	6-Oxogrindelic acid	46.0	230	335^a^	321^a^	Mahmoud et al. (2000)
10	Grindelic acid^b^	50.1	220	321^a^	303^a^	Timmermann et al. (1985)

^a^ [M + H]^+^;

^b^ isolated in this study.

### *G. squarrosa* Extract and Grindelic Acid did Not Show Anti-microbial Activity

The *G. squarrosa* extract (tested at 25–100 μg/ml) and grindelic acid (10–50 µM) did not inhibit bacteria growth. We were not able to detect growth inhibition zone (GIZ, mm) and minimal inhibitory concentration (MIC, μg/mL). The growth inhibition zone was observed only for gram g-positive (*S. pneumoniae* and *S. aureus*) treated with clarithromycin. Obtained results were comparable with reference values present at EUCAST (The European Committee on Antimicrobial Susceptibility Testing) (Bandi and Kompella, 2001).

### *G. squarrosa* Extract Decrease the TLR-4 Expression

**FIGURE 2:**
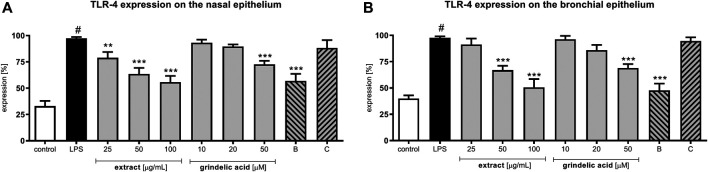
Effects of *G* *. squarrosa* extract and compounds on the TLR-4 expression on the LPS-stimulated: **(A)** nasal epithelial cells (HNEpC) and **(B)** bronchial epithelial cells (NHBE). Data is expressed as the mean ± SEM; three independent experiments assayed in triplicates. Statistical significance ^#^ *p* < 0.01 compared to the non-stimulated control; *p < 0.05, **p < 0.01, ***p < 0.001 compared to stimulated control (+LPS). Control—non-stimulated cells, LPS—LPS-stimulated cells, B—budesonide tested at 50 μM, C—clarithromycin tested at 50 µM.

Stimulation with LPS resulted in a significant increase of TLR-4 expression (Figure 2). Incubation of LPS-stimulated nasal and bronchial epithelial cells with *G. squarrosa* extract resulted in reduction of TLR-4 expression in the presence of all tested concentrations. The obtained results were comparable to budesonide, tested at 50 µM. Budesonide suppressed the TLR-4 expression on nasal epithelial cells to 63.2% and to 48.5% on bronchial epithelial cells (*p* < 0.001). Clarithromycin, as well as grindelic acid affect TLR-4 expression slightly. The tested concentrations, did not damage the cellular membrane integrity of nasal and bronchial epithelial cells as presented in Supplementary Table S1 and Figure S3.

### *G. squarrosa* Extract Modulates Cytokine Production and 65 NF-κb Concentration

**FIGURE 3:**
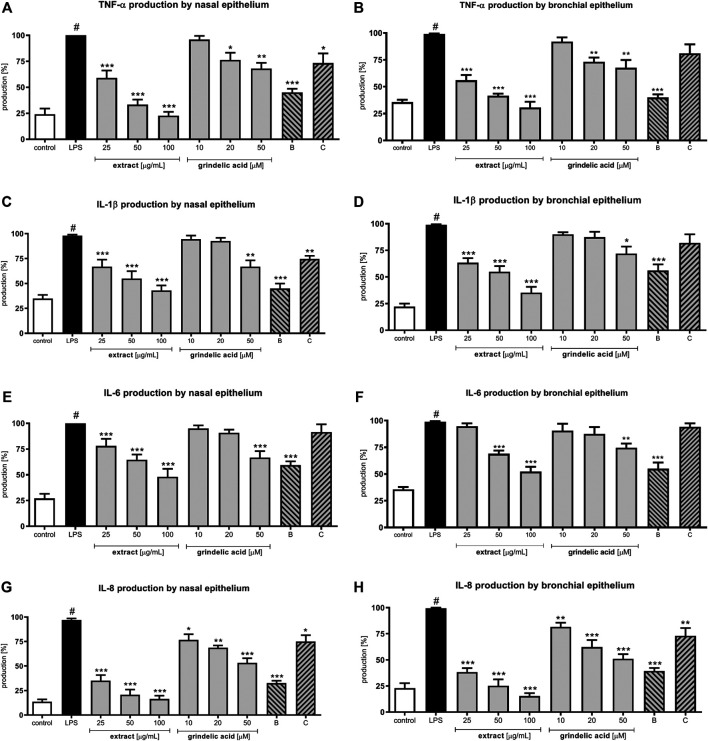
Effects of *G* *. squarrosa* extract and tested compounds on chemo- and cytokines production by LPS-stimulated nasal and bronchial epithelial cells: TNF-α secretion by **(A)** nasal epithelial cells (HNEpC) and **(B)** bronchial epithelial cells (NHBE); IL-1β secretion by **(C)** nasal epithelial cells (HNEpC) and **(D)** bronchial epithelial cells (NHBE); IL-6 secretion by **(E)** nasal epithelial cells (HNEpC) and **(F)** bronchial epithelial cells (NHBE); IL-8 secretion by **(G)** nasal epithelial cells (HNEpC) and **(H)** bronchial epithelial cells (NHBE).

Lipopolysaccharide (LPS) stimulation of human nasal and bronchial epithelial cells resulted in the production wide range of cytokines: TNF-α, IL-1β, IL-6 and IL-8 (leukocyte chemotactic factors). *G. squarrosa* extract as well as grindelic acid were tested at concentration range of 25–100 μg/ml, and 10–50 μM, respectively. IL-1β and TNF-α, called early-released proinflammatory cytokines, were significantly suppressed by *G. squarrosa* extract in both epithelial models (*p* < 0.001; Figures 3A–D). Budesonide, well known corticosteroid with strong anti-inflammatory activity, suppressed IL-1β and TNF-α production, similarly like extract tested at 50 μg/ml (*p* < 0.001). Additionally, *G. squarrosa* extract was more active than clarithromycin, which served as a second positive control (*p* < 0.05). The activity of grindelic acid was significant only in the highest concentration 50 µM (*p* < 0.05) in both epithelial models.

IL-6 and IL-8 were chosen for assessment as late-released proinflammatory cytokines. *G. squarrosa* extract at 25–100 μg/ml significantly inhibited IL-8 production in both epithelial models (*p* < 0.001) in dose dependent manner. The effect of *G. squarrosa* on IL-6 production was significant only up to 50 μg/ml (Figures 3E–H). The biological activity of budesonide was significant (*p* < 0.001) in both epithelial models. However, clarithromycin did not influence on IL-8 and IL-6 production.

**FIGURE 4:**
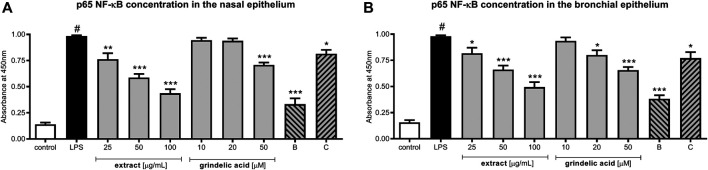
The influence of *G* *. squarrosa* extract and compounds on p65 NF-κB concentration after LPS stimulation of nasal epithelial cells (HNEpC) and **(B)** bronchial epithelial cells (NHBE).

Evaluation of p65 NF-κB concentration explains the molecular mechanism responsible for previously observed cytokine concentration changes. Our data show that *G. squarrosa* extract at the concentration range 25–100 μg/ml significantly decreased p65 production (*p* < 0.001) (Figures 4A,B). Similarly, budesonide tested at 50 µM significantly inhibited p65 production, and final effect was comparable to plant extract tested at 100 μg/ml.

Interestingly, decrease of p65 concentration was observed also for grindelic acid and clarithromycin, both tested at 50 µM.

### *G. squarrosa* Extract Treatment Reduces Macrophage Adhesion to Epithelium

Stimulation with LPS also results in adhesive molecules expression changes (ICAM-1, VCAM-1 and E-selectin), these then direct the emigration of macrophages across the endothelial to epithelial cells. Mentioned adhesive molecules play a different roles depending on the cells where they are located. On the endothelial cells ICAM-1, VCAM-1 and E-selectin precisely coordinate migration, rolling and diapedesis of leukocytes, however, on the epithelial cells they strictly coordinate adhesion leukocytes to epithelium.

The biological effect of *G. squarrosa* treatment was significant only for ICAM-1 expression (Table 3). Comparing to LPS-stimulated control (100%) the plant extract tested at the highest concentration (100 μg/ml) decreased ICAM-1 concentration to 75.3 ± 3.4% in the nasal epithelium, and to 78.2 ± 5.3% in the bronchial epithelium. Similarly, budesonide, tested at 50 µM decreased ICAM-1 expression to 79.4 ± 4.9% and 80.5 ± 3.8%, respectively. Expression of VCAM-1 and E-selectin did not change after treatment.

**TABLE 3:** Effects of *G. squarrosa* extract and tested compounds on the adhesive molecules expression (ICAM-1, VCAM-1 and E-selectin) located on the nasal and bronchial epithelial cells. Data is expressed as the mean ± SEM; three independent experiments assayed in triplicates. Statistical significance ^a^ *p* < 0.01 compared to the non-stimulated control; ^b^ *p* < 0.05, ^c^ *p* < 0.01, ^d^ *p* < 0.001 compared to stimulated control (+LPS).

	ICAM-1 expression [%]		VCAM-1 expression [%]		E-selectin expression [%]	
	Nasal	Bronchial	Nasal	Bronchial	Nasal	Bronchial
Control	34.2 ± 3.6	28.4 ± 1.3	26.4 ± 7.2	30.2 ± 5.5	24.1 ± 3.9	19.3 ± 6.2
Stimulation (+LPS)	100 ± 2.1	100 ± 4.5	100 ± 5.1	100 ± 3.9	100 ± 2.8	100 ± 5.9
G. squarrosa extract + LPS						
25 μg/ml	94.3 ± 5.9	99.3 ± 3.7	89.6 ± 5.3	95.2 ± 3.4	90.9 ± 6.1	97.7 ± 9.4
50 μg/ml	88.3 ± 2.9	90.5 ± 4.2	94.2 ± 2.8	90.3 ± 6.1	94.2 ± 3.1	93.2 ± 1.8
100 μg/ml	75.3 ± 3.4^b^	78.2 ± 5.3^b^	92.5 ± 6.1	88.4 ± 7.9	93.8 ± 4.1	96.2 ± 3.9
Grindelic acid + LPS						
10 µM	89.5 ± 6.9	90.5 ± 8.2	97.4 ± 5.3	98.8 ± 2.4	96.4 ± 2.5	99.3 ± 2.8
20 µM	88.9 ± 9.8	92.1 ± 4.2	95.2 ± 4.6	97.2 ± 1.8	99.4 ± 3.7	93.2 ± 3.7
50 µM	90.4 ± 2.7	91.6 ± 3.7	89.6 ± 4.6	93.2 ± 4.1	91.3 ± 9.2	89.3 ± 7.9
Budesonide 50 µM	79.4 ± 4.9^b^	80.5 ± 3.8^b^	92.5 ± 9.1	90.3 ± 7.1	86.4 ± 6.9	89.5 ± 8.3
Clarithromycin 50 µM	95.3 ± 3.9	92.8 ± 6.2	94.3 ± 5.7	96.3 ± 3.6	99.4 ± 1.8	91.4 ± 6.7

^a^ *p* < 0.01 vs. not stimulated cells;

^b^ *p* < 0.05 vs. LPS-stimulated cells;

^c^ *p* < 0.01 vs. LPS-stimulated cells;

^d^ *p* < 0.001 vs. LPS-stimulated cells.

**FIGURE 5:**
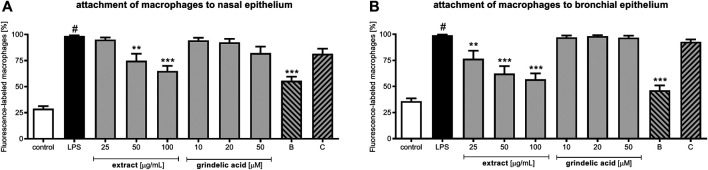
The influence of *G. squarrosa* extract and compounds on adhesion of macrophages to nasal epithelial cells (HNEpC) and bronchial epithelial cells (NHBE).

Continuing, we evaluated influence of extract and compounds on the adhesion of macrophages to both epithelial models (Figure 5). As described in the Material and Methods epithelial cells were previously pre-incubated with extract or compounds, then stimulated with LPS and co-incubated with LPS-stimulated macrophages. Because bacterial infection lead to modulation of pro-inflammatory functions both respiratory epithelium as well as leukocytes, the stimulation with LPS of both models seems to be justified. The proposed cellular models reflect the cellular response to bacterial infection properly.

It is worth to emphasize, that bacterial infection resulting very often with inflammation may lead to excessive activation macrophages. Macrophages are very sensitive for a pro-inflammatory cytokine presence in the bloodstream. Very low concentrations of TNF-α or IL-8 significantly stimulate their chemotaxis to damaged tissues. Because they response to recognized chemoattractant immediately (they play a critical role in nonspecific defense (innate immunity)). Thus, there is a risk of their uncontrolled migration and over-accumulation, what increases the need of pharmacological control their adhesion to epithelium.

Our observations, indicate that *G. squarrosa* extract tested at 50 and 100 μg/ml decreased adhesion of macrophages to nasal epithelium to 74.8 ± 6.7% and to 65.0 ± 8.4%, respectively (Figure 5A). Budesonide, widely used for a treatment of inflammation-related diseases, decreased adhesion to 55.7 ± 5.7%. Similar tendency we observed for the bronchial epithelial cells co-cultured with macrophages (Figure 5B). *G. squarrosa* extract affected the macrophage adhesion to bronchial epithelium much stronger than to nasal epithelium. Tested at concentrations 25, 50, and 100 μg/ml decreased the adhesion to 76.5 ± 9.1, 62.3 ± 7.7, and 56.8 ± 5.1%. Comparing the strong anti-inflammatory activity of budesonide (decreased adhesion of macrophages to bronchial cells to 46.3 ± 8.5%) with anti-inflammatory properties of tested *G. squarrosa* extract and its lack of cytotoxic properties (Supplementary Table S1) our findings proved competitiveness of phytotherapy.

### *G. squarrosa* Extract and Grindelic Acid Modulate Pro- and Anti-inflammatory Functions of LPS-Stimulated Macrophages

The *G. squarrosa* extract (at 25–100 μg/ml) significantly upregulated IL-10 receptor expression. Similar effect was observed for grindelic acid tested at 50 µM (*p* < 0.05) (Table 4). The extract and grindelic acid significantly promote anti-inflammatory functions of macrophages through stimulation of TGF-β production (*p* < 0.001). *G. squarrosa* extract treatment also resulted in suppression of pro-inflammatory cytokines: TNF-α and IL-6 in dose dependent manner (*p* < 0.001 for all tested concentrations). Budesonide, well known anti-inflammatory drug capable of suppressing cytokine release, at 50 µM inhibited TNF-α and IL-6 to 45.4 and 56.5%, respectively. The activity of clarithromycin was evident but more modest. The tested extract, compounds and solvent (DMSO) were not toxic for mononuclear, as shown by assessing their influence on monocyte/macrophages membrane integrity at the tested concentrations (Supplementary Table S1).

**TABLE 4:** Effects of *G. squarrosa* extract and tested compounds on pro- and anti-inflammatory functions of LPS-stimulated monocytes/macrophages: IL-10 receptor expression on the surface of stimulated macrophages; TGF-β secretion by stimulated macrophages [pg/mL]; IL-6 secretion by stimulated macrophages [%]; TNF-α secretion by stimulated macrophages [%]. Data is expressed as the mean ± SEM; three independent experiments assayed in triplicates. Statistical significance ^a^ *p* < 0.01 compared to the non-stimulated control; ^b^ *p* < 0.05, ^c^ *p* < 0.01, ^d^ *p* < 0.001 compared to stimulated control (+LPS).

	Surface IL-10 receptor expression [%]	TGF-β release [pg/mL]	IL-6 release [%]	TNF-α release [%]
Control	100.2 ± 21.6	33.7 ± 7.9^a^	34.4 ± 4.2	24.3 ± 5.4
Stimulation (+LPS)	46.4 ± 15.5^a^	8.5 ± 4.8^a^	100.0 ± 2.1^a^	100.4 ± 3.3^a^
G. squarrosa extract + LPS				
25 μg/ml	257.4 ± 61.8^d^	37.5 ± 2.1	64.3 ± 6.2^d^	58.5 ± 2.5^d^
50 μg/ml	476.6 ± 62.4^d^	43.6 ± 4.8	60.2 ± 4.4^d^	55.3 ± 3.8^d^
100 μg/ml	904.3 ± 96.4^d^	58.4 ± 4.4^c^	52.5 ± 2.2^d^	45.2 ± 2.8^d^
Grindelic acid + LPS				
10 µM	13.8 ± 3.6	n.d	87.9 ± 4.7	90.4 ± 3.3
20 µM	16.5 ± 5.5	n.d	84.4 ± 8.2	85.3 ± 2.7
50 µM	18.1 ± 6.2	35.3 ± 9.8	74.3 ± 3.5^c^	76.6 ± 0.9
100 μg/ml				
Budesonide 50 µM	50.0 ± 5.6	40.3 ± 2.1	56.4 ± 3.8^d^	65.9 ± 1.6^d^
Clarithromycin 50 µM	64.4 ± 4.7	n.d	68.4 ± 6.6^c^	78.6 ± 2.1^b^

n.d., not detectable.

^a^ *p* < 0.01 vs. not stimulated cells;

^b^ *p* < 0.05 vs. LPS-stimulated cells;

^c^ *p* < 0.01 vs. LPS-stimulated cells;

^d^ *p* < 0.001 vs. LPS-stimulated cells.

## Discussion

Aerial parts of *Grindelia* were traditionally used to treat cold-related diseases. Importantly, as liquid extracts and syrups they are still widely distributed at Polish and German markets (Gierlikowska et al., 2020a). The commercial spread of extract for cough and bronchitis treatment increased the need of understanding the biological mechanism of action.

Although the phytochemical composition of *Grindelia squarrosa* is well-known (Veres et al., 2014; Nowak et al., 2019) the biological activity is still not explained. In our previous study we documented anti-inflammatory properties of *G. squarrosa* extract tested on human neutrophilic model (Gierlikowska et al., 2020a). Now, we expand the study and present how *G. squarrosa* regulates pro-inflammatory functions of respiratory epithelium and macrophages, and how modulates their mutual interactions. Our main findings explain how *G. squarrosa* extract modulates molecular mechanisms involved in epithelial response to LPS stimulation, and how it regulates migration and adhesion of macrophages to respiratory epithelium.

The acute episode of a cold starts with virus infection and may be accompanied by bacterial superinfection. *Grindelia* species were shown to exert antimicrobial activity, especially against gram negative *E. coli* (Hassan et al., 2014). According to Hassan et al. (Hassan et al., 2014) the extract of *G. squarrosa* flowers showed inhibition diameters in excess of 19.3 mm of *A. caviae*, *M. luteus* and *P. alvei*. Similar results were obtained by Jacobs et al. (Jacobs et al., 2010). Using different extracts solvents (CCl_4_ and 1-BuOH) they confirmed anti-microbial properties of *G. squarrosa*: CCL_4_-extract inhibited *E. coli*, *S. aureus* and *C. albicans* growth, whereas 1-BuOH-extract inhibited *S. aureus*, *P. aeruginosa* and *K. pneumoniae* (Jacobs et al., 2010). In the present study we tested anti-microbial activity of 60%-EtOH extract, and we did not observe any anti-microbial activity. Only clarithromycin, used as positive control, significantly inhibited growth of selected gram positive: *S. aureus* and *S. pneumoniae* (growth inhibition zone, GIZ: 30–34 mm). The water-ethanol extraction, as we performed in present study, reflects the way in which market preparations are obtained. Comparing our results with experiments performed by Hassan (Hassan et al., 2014) and Jacobs (Jacobs et al., 2010) we could hypothesize that different solvents may determine differences in the phytochemical composition and affect on their anti-microbial properties.

The pathogenic infection results in development of full-symptomatic inflammation (Roxas and Jurenka, 2007). Bacterial infection starts with interaction of pathogens with Toll-like receptors located on epithelial cells. To response to gram negative bacteria/LPS, TLR-4 expression increases. To our knowledge, there is no data showing the influence of *G. squarrosa* extract and/or grindelic acid on TLR-4 expression. Using two cellular models (nasal and bronchial epithelium) we observed significant decrease of TLR-4 expression after treatment. Modest activity of grindelic acid may suggest, that anti-inflammatory activity of the extract is not determined be grindelic acid (being the major diterpene), but it is a synergistic effect of all compounds present in the extract.

When nasal and bronchial epithelial cells are exposed to bacterial infection, they secrete a wide spectrum of pro-inflammatory cytokines in a precisely defined sequence (Broekman et al., 2016). At the beginning of infection, low amounts of “early-inflammatory cytokines”—IL-1β and TNF-α are released. Both cytokines intensify production of the “late-inflammatory cytokines”—IL-6 and IL-8 by the respiratory epithelial cells (Broekman et al., 2016), and stimulate chemotaxis of macrophages. It is known that cytokines promote adequate immune response, but prolonged cytokines production by stimulated respiratory epithelial cells may exacerbate inflammation. The side effects arise from excessive macrophage chemotaxis and prolonged stimulation of their pro-inflammatory functions.

Our next finding indicates that *G. squarrosa* extract significantly inhibited IL-1β, TNF-α, IL-6, and IL-8 production by epithelial cells *via* p65 NF-κB suppression. Our observations are coincident with observations performed by La et al. (La et al., 2010). La and al. presented anti-inflammatory properties of *Grindelia robusta* Nutt. tested on monocyte-derived macrophages (U937, human monoblastic leukemia cell line) (La et al., 2010). The authors showed that *G. robusta* extract tested at concentration range 25–100 μg/ml inhibited dose-dependently the secretion of IL-6, MCP-1 and, to a lesser extent, PGE2 and TNF-α. Such inhibition was also observed for MMP-1, -3, -7, -8, -9, and -13 production. The ability of *G. robusta* extract to reduce the LPS-induced secretion of inﬂammatory mediators and MMPs was associated with a reduction of nuclear factor-kappa B (NF-kB) p65 activation (La et al., 2010). Despite differences in chemical composition of *G. squarrosa* and *G. robusta* it is worth emphasizing that both extracts present ability to suppression of pro-inflammatory cytokine production (La et al., 2010; Gierlikowska et al., 2020a).

Secretion of proinflammatory chemokines and cytokines (interleukins IL-8, IL-1β and TNF-α) induces inflammatory and immune responses (Liu et al., 2016). In respiratory epithelium these effects are associated with the activation of mitogen-activated protein kinases (MAPKs), i.e., p38 kinase, p42/44 extracellular signal-regulated kinase (ERK), and c-Jun NH_2_-terminal kinases (JNKs), and are regulated by two major transcription factors families, i.e., NF-κB and AP-1 (Liu et al., 2016; Yang et al., 2017; Zhang et al., 2018). In the available literature there is no data regarding the influence of *G. squarrosa* extract and grindelic acid on MAPK pathway and AP-1. However, based on results obtained for p65 NF-κB we could hypothesize that *G. squarrosa* may modulate p65 NF-κB *via* inhibition of MAPKs activity. The activation of AP-1 is also mediated by MAPKs thus verification of mentioned hypothesis may highlight new targets for a cold-related diseases treatment.

Based on our experiments performed on a two independent epithelial models (nasal and bronchial epithelium), we can speculate that *G. squarrosa*, through strong inhibition of chemoattractant production (IL-1β, TNF-α, IL-6, and IL-8), may potentially limit activation, and migration macrophages to damaged epithelial cells. However, macrophages are very sensitive to cytokines released to bloodstream, thus even small amounts of secreted cytokines may activate adequate immune response.

The migration of macrophages to damaged epithelium is strictly dependent on TNF-α, IL-β, and IL-8 concentration in the bloodstream (Rosseau et al., 2000). Released by damaged epithelium pro-inflammatory cytokines increase expression of VCAM-1, E-selectin, and ICAM-1 (Turner et al., 2011). On the contrary to VCAM-1 and E-selectin, ICAM-1 appears on the epithelium later and is located only at the site of inflammation.

In our study, we tested influence of *G. squarrosa* on the expression of mentioned above adhesive molecules, however, we did not observe influence of extract on VCAM-1 and E-selectin expression. At the highest concentration (100 μg/ml) *G. squarrosa* extract slightly inhibited only ICAM-1 expression. The comparable effect was noticed for budesonide tested at 50 µM. The reduction of ICAM-1 expression may be related with inhibition of cytokine production. As therapeutic target for ICAM-1 suppression were identified IL-8 and TNF-α (Kwon et al., 2011; Kim et al., 2013; Gao et al., 2018). Why extract modulates only adhesion molecule responsible for attachment of leukocytes to epithelium, also remains to be elucidated.

Although, *G. squarrosa* treatment did not affect on epithelial adhesive molecules expression directly, it suppressed the macrophage adhesion to epithelium. At concentration above 50 μg/mL *G squarrosa* significantly inhibited the adhesion of macrophages to both epithelial models. The lowest tested concentration (25 μg/ml) decreased adhesion of macrophages only to bronchial epithelium.

The differences between an affect of *G. squarrosa* treatment on percentage of adhered of macrophages to nasal and bronchial epithelium may be partially explained by epithelial cells anatomical location, predispose to infection/damage and ability to regeneration. The nasal cavity is constantly inhabited by pathogens and is more exposed to mechanical damage (Man et al., 2017). The lower respiratory tract is sterile, thus bronchial epithelial cells must have developed mechanisms that protect not only against pathogens, but also against excessive accumulation of phagocytes. Resseau et al. (Rosseau et al., 2000) explained the stricted regulation of emigration into the alveolar compartment by the difficulties with successful clearance of macrophages from the lung parenchyma. Thus, the observed stronger inhibition of macrophage adhesion to bronchial epithelium then nasal epithelium may be a result of an additive effect of *G. squarrosa* treatment and physiological response of bronchial cell to interactions with macrophages.

At the end, we confirmed that *G. squarrosa* extract stimulate anti-inflammatory functions of macrophages by increase of TGF-β production and IL-10 expression what accelerates the removal of pathogens and damaged tissues (Aggarwal et al., 2014). *G. squarrosa* also reduced concentration of pro-inflammatory cytokines (IL-6 and TNF-α) released by macrophages, successfully reducing inflammation.

In our opinion the biological activity of grindelic acid was a type of cell-dependent. At the highest tested concentration (50 µM) grindelic acid selectively modulated respiratory epithelium functions (TLR-4 expression, cytokine production and p65 NF-κB concentration). Interestingly, grindelic acid did not affect adhesive molecules expression and pro-inflammatory functions of macrophages. According to Leiva-Juárez et al. (Leiva-Juárez et al., 2018) the highly plastic epithelial barrier responds to detected threats *via* modulation of drug accumulation, paracellular flux, intercellular communications, mucin production, and periciliary fluid composition. Epithelial stimulation induces production of cytokines that recruit and sculpt leukocyte-mediated responses, and promotes epithelial generation of antimicrobial effector molecules that are directly microbicidal. Thus epithelium can alternately enhance tolerance to pathogens, preventing tissue damage through induced inhibitory signals and through potential accumulation of compound may attenuate of injurious leukocyte responses (Leiva-Juárez et al., 2018).

**FIGURE 6:**
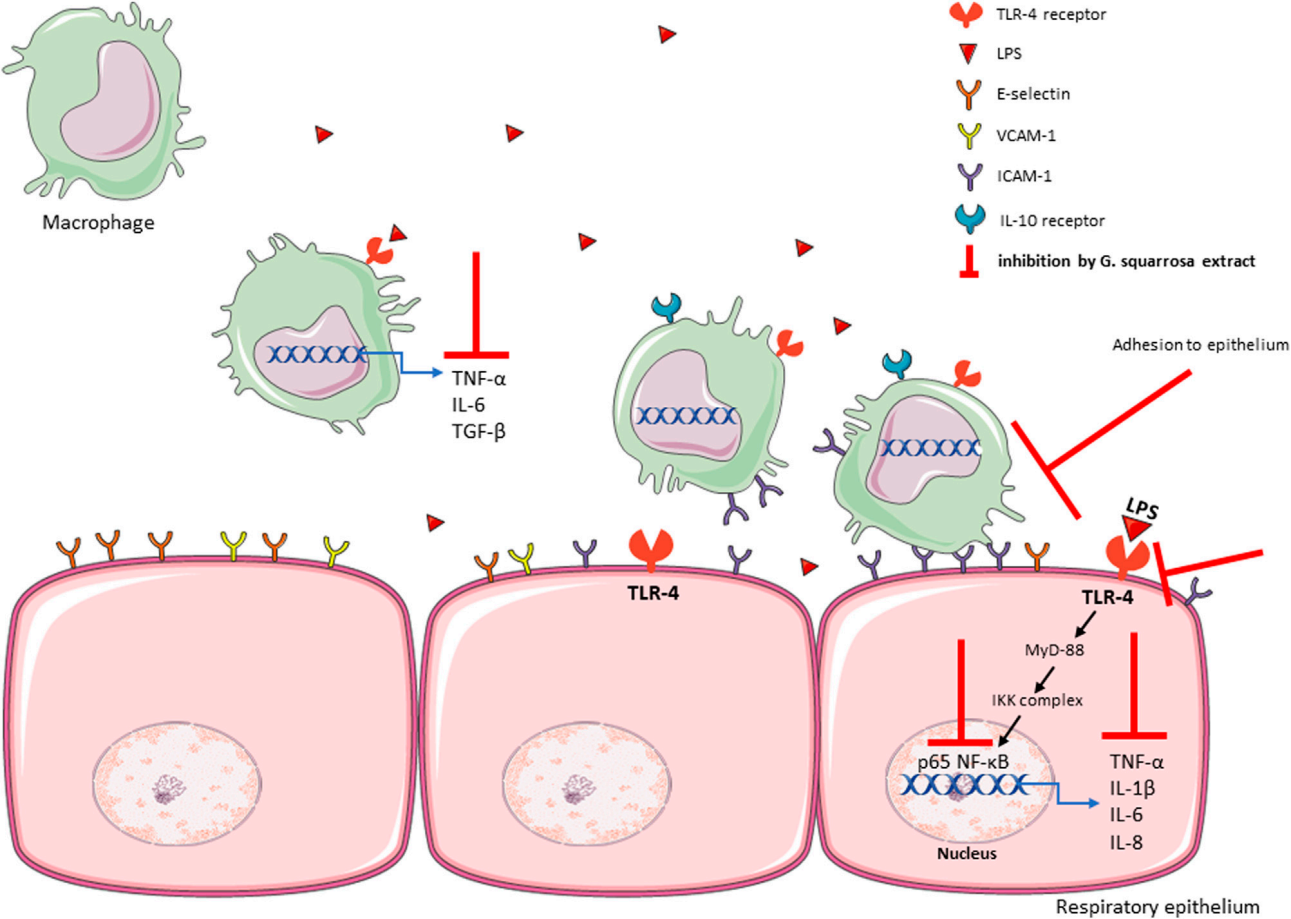
The summary of influence of *G. squarrosa* extract on the proinflammatory functions of respiratory epithelium.

Summarizing, our results explain how *G. squarrosa* modulates pro-inflammatory functions of nasal, bronchial epithelium and macrophages, and their mutual interactions (Figure 6). We confirmed anti-inflammatory properties of *G. squarrosa* and influence on modulation of leukocyte adhesion to nasal and bronchial epithelial cells. The obtained results allow to better understand the therapeutic efficacy of *G. squarrosa* raw material.

## Conclusion

The present study justified the traditional use of *Grindelia squarrosa* aerial parts for treatment of cold-related diseases. The wide-spread use of *G. squarrosa* in the traditional medicine and effectiveness during cold-related diseases treatment may be explained by modulation of pro-inflammatory functions respiratory epithelium and macrophages.

The observed suppression of inflammation was a result of “cascade suppressed pro-inflammatory cell response.” The *G. squarrosa* suppressed TLR-4 expression, decreased p65 NF-κB concentration and resulted in suppression of TNF-α, IL-1β, IL-6, and IL-8 production. Moreover, we noticed stimulation of anti-inflammatory functions of macrophages and inhibition of their adhesion to nasal and bronchial epithelium. Our observations suggest the high therapeutic potential of *G. squarrosa* extract during symptomatic treatment of cold-related diseases.

## Data Availability Statement

The original contributions presented in the study are included in the article/Supplementary Material, further inquiries can be directed to the corresponding author.

## Ethics Statement

The studies involving human participants were reviewed and approved by Committee on Bioethics at the Medical University of Warsaw. The patients/participants provided their written informed consent to participate in this study.

## Author Contributions

BG designed the study and supervised the work. BG and AK selected plant material and obtained financial support (FW25/PM3/18). DK and AK performed phytochemical analysis. BG, AF, WG, and JS planned and performed the biological experiments and carried out data analysis. BG wrote the manuscript. UD critically reviewed the manuscript.

## Funding

The study was financed by Medical University of Warsaw, Poland—program: Young Researcher Grant (No. FW25/PM3/18).

## Conflict of Interest

The authors declare that the research was conducted in the absence of any commercial or financial relationships that could be construed as a potential conflict of interest.
