# Supplementary material for: Grindelia squarrosa Extract and Grindelic Acid Modulate Pro-inflammatory Functions of Respiratory Epithelium and Human Macrophages
Source: Front Pharmacol. 2021 Jan 18;11:534111. doi: 10.3389/fphar.2020.534111 (PMC7848105; doi:10.3389/fphar.2020.534111)

**SUPPORTING INFORMATION**

***Grindelia squarrosa* extract and grindelic acid modulate pro-inflammatory functions of respiratory epithelium and human macrophages**

Barbara Gierlikowska^1^*, Agnieszka Filipek^2^, Wojciech Gierlikowski^3^, Dominika Kania^2^, Joanna Stefańska^4^, Urszula Demkow^1^, Anna K. Kiss^2^

^1^Department of Laboratory Diagnostics and Clinical Immunology of Developmental Age, Medical University of Warsaw, Żwirki i Wigury 63a , 02-091 Warsaw, Poland

^2^Department of Pharmacognosy and Molecular Basis of Phytotherapy, Medical University of Warsaw, Banacha 1, 02-097 Warsaw, Poland

^3^Department of Internal Medicine and Endocrinology, Medical University of Warsaw, Banacha 1a, 02-097 Warsaw, Poland

^4^Department of Pharmaceutical Microbiology, Medical University of Warsaw, Banacha 1b, 02-097 Warsaw, Poland

*Correspondence: Barbara Gierlikowska, Department of Laboratory Diagnostics and Clinical Immunology of Developmental Age, Medical University of Warsaw, Żwirki i Wigury 63a, 02-091 Warsaw, Poland, barbara.gierlikowska@wum.edu.pl

**Figure S1.** Scheme of isolation of grindelic acid (GA) from *Grindelia squarrosa* extract.

**
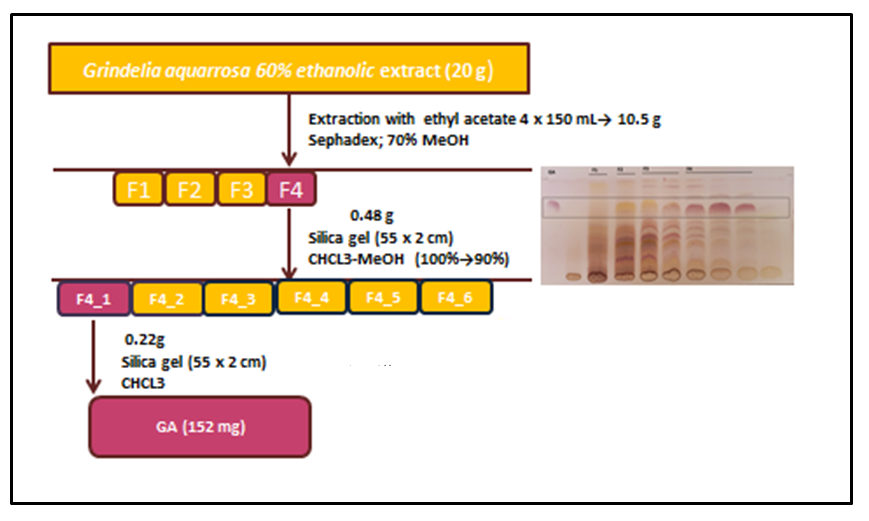
**

**Figure S2.** C-NMR data of grindelic acid.

**
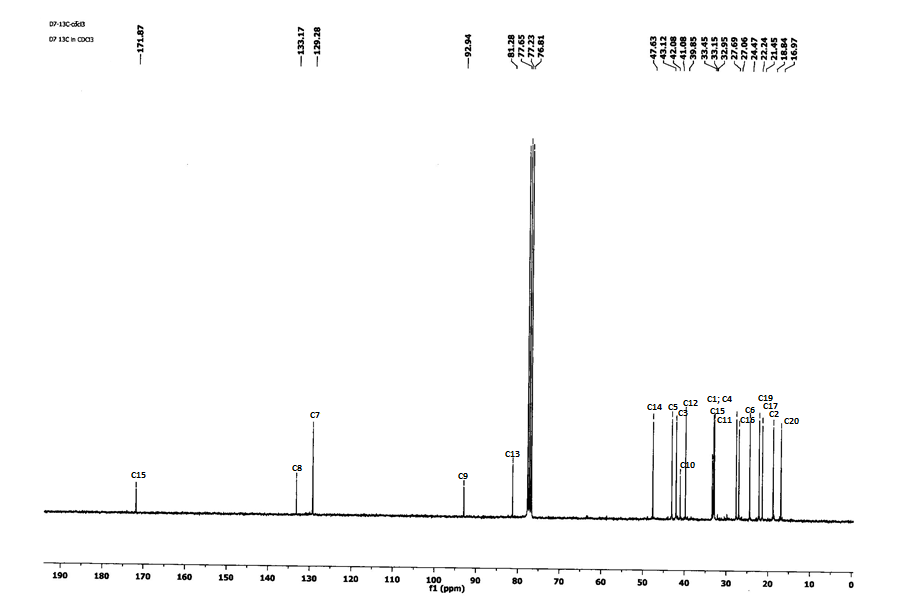
**

**Table S1**.The influence of *G. squarrosa* extract, grindelic acid and positive controls (budesonide, clarithromycin) on cells membrane integrity. Results are presented as average ± SEM.

**Cytotoxicity (propidium iodide staining positive cells [%])**

|  | **Death cells [%]** | | |
| --- | --- | --- | --- |
|  | macrophages | HNEpC | NHBE |
| control | 9.3 ± 2.2 | 7.2 ± 1.4 | 6.4 ± 3.2 |
| LPS | 13.8 ± 1.2 | 9.2 ± 1.5 | 8.5 ± 2.6 |
| **Grindelia squarrosa extract** (+LPS) | | |  |
| 25 µg/mL | 9.1 ± 1.8 | 8.7 ± 1.2 | 11.4 ± 2.1 |
| 50 µg/mL | 10.1 ± 1.2 | 9.5 ± 0.7 | 10.3 ± 1.7 |
| 100 µg/mL | 12.8 ± 2.3 | 14.8 ± 1.8 | 12.5 ± 3.9 |
| **Grindelic acid** (+LPS) | | |  |
| 10 µM | 11.6 ± 7.4 | 9.3 ± 2.4 | 10.4 ± 3.2 |
| 20 µM | 12.5 ± 3.5 | 10.4 ± 1.6 | 9.8 ± 2.5 |
| 50 µM | 12.4 ± 1.2 | 10.2 ± 1.3 | 9.5 ± 2.7 |
| Budesonide 50 µM (+LPS) | 14.4 ± 2.1 | 12.5 ± 1.9 | 10.3 ± 1.8 |
| Clarithromycin 50 µM (+LPS) | 17.3 ± 2.1 | 13.3 ± 0.5 | 9.4 ± 2.4 |

**Figure S3.** The influence of G. squarrosa extract and grindelic acid on cell viability. Data express [%] of dead cells.


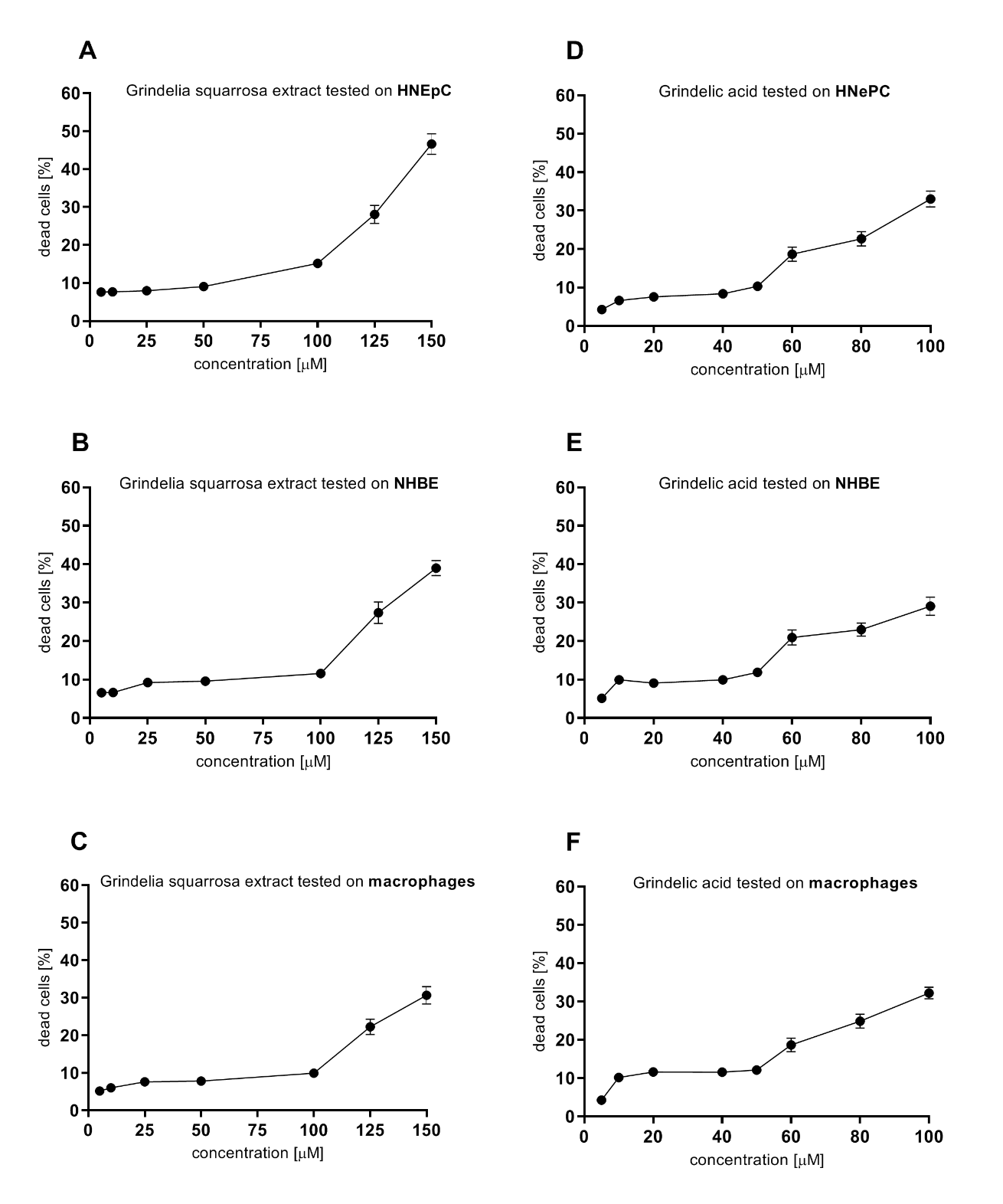

Supplement: Supplementary file 1 [file datasheet1.docx]
